# Comparative effectiveness of sodium-glucose co-transporter 2 inhibitors for controlling hyperglycaemia in patients with type 2 diabetes: protocol for a systematic review and network meta-analysis

**DOI:** 10.1136/bmjopen-2015-010252

**Published:** 2016-01-29

**Authors:** Min Chen, Chun-Guang Xie, Hong Gao, Hui Zheng, Qin Chen, Jian-Qiao Fang

**Affiliations:** 1 Clinical Department, Teaching Hospital of Chengdu University of Traditional Chinese Medicine, Chengdu, Sichuan, China; 2 Acupuncture & Tuina College/The Third Teaching Hospital, Chengdu University of Traditional Chinese Medicine, Chengdu, Sichuan, China; 3 The Third Clinical College of Zhejiang Chinese Medical University, Hangzhou, Zhejiang, China

**Keywords:** SGLT2 inhibitors, hyperglycemia, network meta-analysis, study protocol

## Abstract

**Introduction:**

As a new class of glucose-lowering drugs, sodium-glucose co-transporter 2 (SGLT2) inhibitors are effective for controlling hyperglycaemia, however, the relative effectiveness and safety of 6 recently available SGLT2 inhibitors have rarely been studied. Therefore, we aim to perform pairwise comparisons of the 6 SGLT2 inhibitors.

**Methods and analysis:**

A systematic review and network meta-analysis will be conducted. Clinical studies that examine effectiveness and safety of either canagliﬂozin, dapagliﬂozin, empagliﬂozin, ipragliflozin, tofogliflozin or luseogliflozin will be included. These studies will be systematically retrieved in MEDLINE, EMBASE and the Cochrane Library, from inception to November 2015. Two reviewers will independently screen for eligible studies and then extract data from the studies as well as assess risk of bias. Discrepancies in screening and data extraction will be arbitrated by a third reviewer. A traditional meta-analysis will be performed to combine the effect sizes calculated from head-to-head comparisons with a random effect model. The effect sizes computed from indirect comparisons will be further combined in a network meta-analysis. Heterogeneity will be tested with the Cochrane's Q statistic, and publication bias will be assessed using a funnel plot and the Egger's test.

**Ethics and dissemination:**

Relative effectiveness and harms of the 6 SGLT2 inhibitors will be demonstrated through this systematic review and network meta-analysis. The result of the review will be disseminated through a peer-review journal and conference presentations. Patients, clinicians and policymakers will benefit from this review in selecting a SGLT2 inhibitor for glucose control in patients with type 2 diabetes.

**Trial registration number:**

PROSPERO CRD42015025981.

**Strengths and limitations of this study**

- We will include recently published studies that assessed incidence of cardiovascular disease, ketoacidosis and cancer caused by SGLT2 inhibitors, which will add knowledge to the safety of SGLT2 inhibitors.
- The result of this meta-analysis will help patients with type 2 diabetes, clinicians and policymakers in selecting a SGLT2 inhibitor for controlling hyperglycaemia.
- A possible limitation is that we may not have enough data to perform pairwise comparisons between the SGLT2 inhibitors, since these inhibitors will be compared in four situations: monotherapy, dual therapy, triple or quadruple therapy and in combination with insulin.

## Introduction

Hyperglycaemia is a major manifestation of diabetes mellitus. The most important biomarker of hyperglycaemia is glycated haemoglobin (HbA1c). Including HbA1c to the diagnostic criteria accounts for a 75% increase of individuals with diabetes mellitus across all age-groups.^1^ Patients with elevated HbA1c level are at high risk for developing diabetic retinopathy and cardiovascular disease.^2–4^ Lowering HbA1c to <7.0% significantly reduces the risk of microvascular complications in patients with type 2 diabetes.^5–7^ Given that type 2 diabetes is, globally, a major public health problem (affecting 347 million individuals in the year 2008),^8^ stringent control for hyperglycaemia is needed.

As a new class of drugs, sodium-glucose co-transporter 2 (SGLT2) inhibitors are recommended in a report on hyperglycaemia management released by the American Diabetes Association (ADA) and the European Association for the Study of Diabetes (EASD).^9^ SGLT2 inhibitors activate at the proximal nephron to decrease glucose absorption, so they are independent of insulin and therefore can be used in any stage of type 2 diabetes. Several systematic reviews have shown that SGLT2 inhibitors are effective for controlling HbA1c.^10–17^ In these reviews, when different doses of a SGLT2 inhibitor are tested in a trial, only the highest dose of this SGLT2 is chosen to include for meta-analysis. In addition, some reviews summarise canagliﬂozin, dapagliﬂozin and empagliﬂozin in the same category, and assess them as one treatment, ignoring heterogeneity in their treatment effects.^13^ ^14^ Rosenstock *et al*^18^ found that 50 mg canagliflozin worked better than 200 mg canagliflozin in lowering HbA1c. A similar finding of dose-ranging effect of dapagliflozin was discovered in a systematic review.^12^ Therefore, we hypothesise that the treatment effects of canagliﬂozin, dapagliﬂozin and empagliﬂozin are different, especially when administered in different doses. Recently, three new SGLT2 inhibiting drugs (ipragliflozin, tofogliflozin and luseogliflozin) were introduced to clinical practice and tested by randomised controlled trials,^19–21^ but they were not included in previous systematic reviews. A systematic review protocol was recently published to evaluate the efficacy of SGLT2 inhibitors by comparing them to placebo.^22^ However, this systematic review did not assess the efficacy of ipragliflozin, tofogliflozin and luseogliflozin, nor did it assess their relative effectiveness. Additionally, adverse events of the 6 SGLT2 inhibitors have not been fully evaluated in previous reviews, especially for events such as cardiovascular diseases, ketoacidosis and cancer.

Methods of network meta-analysis (NMA) have been developed as alternative treatment options for disease conditions, however, increased and comparative effectiveness research is needed. NMA can be carried out using frequentist or Bayesian statistics.^23^ Lumley developed a package, ‘NLME’, for conducting NMA in a frequentist framework,^24^ with a major advantage of addressing inconsistency in the network to assess the uncertainty in treatment estimates. However, the ‘NLME’ package could not handle a trial with three or more arms. As the techniques of NMA develop, Rucker has proposed a new NMA statistical solution to deal with trials that have three or more arms,^25^ with the advantage of addressing inconsistency within and between trials as well as adjusting treatment estimates in trials with multiple arms. Therefore, we aim to test the relative effectiveness and safety of canagliﬂozin, dapagliﬂozin, empagliﬂozin, ipragliflozin, tofogliflozin and luseogliflozin, using a systematic review and NMA in a frequentist framework.

## Methods

A systematic review and network meta-analysis will be performed. In the systematic review, we will retrieve clinical studies that tested the effectiveness and safety of canagliﬂozin, dapagliﬂozin, empagliﬂozin, ipragliflozin, tofogliflozin and luseogliflozin. After evaluating the eligibility of potential studies, we will collect data from them and assess risk of bias. In the network meta-analysis, we will combine both—direct and indirect comparisons—using a statistical package of ‘netmeta’ which has been designed for pairwise comparisons in a frequentist framework. This study has been registered at PROSPERO (http://www.crd.york.ac.uk/PROSPERO) with registration number CRD42015025981.

## Data source

We will search for studies that examined effectiveness and adverse events of the 6 SGLT 2 inhibitors in the following electronic databases: MEDLINE (via Ovid), EMBASE ( http://www.embase.com) and the Cochrane library, from inception to November 2015. Table 1 shows a search strategy developed with comprehensive use of medical subject headings and keywords. Besides the electronic search, we will also perform a manual search for conference abstracts or e-posters in the areas of diabetes, in the ADA, EASD, Canadian Diabetes Association (CDA) and International Diabetes Federation (IDF). Studies that were reported in the conference abstracts or e-posters will be summarised in a narrative review and excluded from a subsequent meta-analysis. We will also search websites (clinicaltrials.gov, anzctr.org.au, chictr.org and http://www.isrctn.com) for registration records to identify clinical studies that meet our inclusion criteria, and the results will also only be presented in the narrative review. Additionally, we will screen the reports used to support the National Institute for Health and Care Excellence (NICE) appraisals of dapagliflozin, canagliflozin and empagliflozin (available at https://www.nice.org.uk/guidance/indevelopment/gid-tag471). The result of our literature search will be compared with results of previous systematic reviews that evaluate the effectiveness of SGLT2 inhibitors, to ensure that we have all the eligible studies. Language restrictions will not be applied in this systematic review.

**Table 1:** Search strategy for MEDLINE (via OVID)

No.	Search terms
1	randomized controlled trial.pt.
2	controlled clinical trial.pt.
3	randomized.ab.
4	randomised.ab.
5	placebo.ab.
6	randomly.ab.
7	trial.ab.
8	groups.ab.
9	1 or 2 or 3 or 4 or 5 or 6 or 7 or 8
10	exp hyperglycemia/
11	exp Diabetes Mellitus, Type 2 /
12	hyperglycemia. ti, ab.
13	Type 2 diabetes. ti, ab.
14	10 or 11 or 12 or 13
15	sodium glucose co-transporter. ti, ab.
16	SGLT2. ti, ab.
17	Canagliflozin. sh, ti, ab.
18	Dapagliflozin. sh,ti, ab.
19	Empagliflozin. sh, ti, ab.
20	ipragliflozin. sh, ti, ab.
21	tofogliflozin. sh, ti, ab.
22	luseogliflozin. sh, ti, ab.
23	A10BX11. sh, ti, ab.
24	A10BX09. sh, ti, ab.
25	A10BX12. sh, ti, ab.
26	ASP1941. sh, ti, ab.
27	CSG452. sh, ti, ab.
28	TS-071. sh, ti, ab.
29	15 or 16 or 17 or 18 or 19 or 20 or 21 or 22 or 23 or 24 or 25 or 26 or 27 or 28
30	9 and 14 and 29

SGLT2, sodium-glucose co-transporter 2 inhibitor. A10BX11, A10BX09, A10BX12, ASP1941, CSG452 and TS-071 are the codenames for canagliflozin, dapagliflozin, empagliflozin, ipragliflozin, tofogliflozin and luseogliflozin, respectively.

## Criteria for screening studies

### Study design

We will include clinical studies (including randomised controlled trials (RCTs) and observational studies) that assess effectiveness and safety of the 6 SGLT2 inhibitors, considering that adverse events were assessed in longer term observational studies,^26^ ^27^ especially events of cardiovascular disease, ketoacidosis and cancer. We will exclude trials of a crossover design for the nature of the primary outcome. We will not exclude RCTs according to methods of blinding. However, we will categorise the RCTs (open label, single blind or double blind) according to the blinding methods and run a sensitivity analysis with exclusion of trials without blinding.

### Participants

We will include participants aged over 18 years and diagnosed with type 2 diabetes^28^; the HbA1c level of the participants will need to be over 7% (53.0 mmol/mol).^9^ Since pharmacokinetic parameters are slightly altered in the case of mild chronic kidney disease (CKD)^29^ after SGLT2 inhibitors are used, we will exclude trials that include participants with moderate to severe CKD. History of taking medication and duration of diabetes will not be restricted. The duration of diabetes will be classified into three categories: less than 2 years, 3–9 years and >10 years from diagnosis.^17^ This classification will be used in a subgroup analysis. Ipragliflozin, tofogliflozin and luseogliflozin have been tested mainly in the Japanese population, which may introduce heterogeneity in the overall analysis, so we will run a subgroup analysis with inclusion of only trials that test these three drugs.

### Interventions and comparisons

Canagliﬂozin, dapagliﬂozin, empagliﬂozin, ipragliflozin, tofogliflozin and luseogliflozin can be used as monotherapy, dual therapy, triple or quadruple therapy and in combination with insulin. Background glucose-lowering drugs will be restricted to: metformin, insulin, sulfonylurea, dipeptidyl peptidase-4 (DDP-4) inhibitor.^9^ These six drugs should be compared with placebo or antidiabetic medications, being administered orally or intravenously. We will include studies with these daily doses: canagliflozin, from 50 to 300 mg; dapagliflozin, from 2.5 to 20 mg; empagliflozin, from 5 to 25 mg; ipragliflozin, from 12.5 to 100 mg; tofogliflozin, from 10 to 40 mg; luseogliflozin, from 0.5 to 5 mg. Given the insulin-independent mechanism of action and the result of a previous systematic review,^17^ we will include trials that have a minimum treatment duration of at least 12 weeks.

## Outcome measurements

The primary outcome of this study will be the absolute change in HbA1c (%) compared with baseline. The secondary outcomes will include proportion of participants achieving the HbA1c target <7%,^28^ mean change in body weight from baseline, mean change in blood pressure from baseline and incidence of adverse events (urinary and genital tract infections, cardiovascular disease, ketoacidosis and cancer). SGLT2 inhibitors have proven to have an effect on reducing body weight,^9^ which is beneficial for patients with type 2 diabetes, so we will assess the change in body weight. Urinary and genital tract infections are the most frequent side effects from using SGLT2 inhibitors,^9^ and cardiovascular disease, ketoacidosis and cancer have recently been evaluated,^30–33^ so we will focus on these harms. Additionally, whether SGLT2 inhibitors should be used in patients with diabetes with impaired renal function is still controversial,^34^ ^35^ so we will evaluate the change in estimated glomerular filtration rate.

## Methods of study selection and data extraction

Two reviewers (MC and HG) will independently screen titles and abstracts for eligible studies after the literature search. And they will further screen full-text copies if they cannot decide on the basis of the titles and abstracts. Discrepancies in eligibility of the studies will be solved by discussion and arbitrated by a third reviewer (C-GX). Data extraction from the eligible studies will be independently performed by the two reviewers (MC and HG). The data will include general information, population and settings, methods, participants, interventions, outcomes and results. The general information will include first author, funding source, and type and year of publication. For the population and settings, we will collect information about the country the clinical study was initiated in, diagnostic criteria used to identify type 2 diabetes and recruitment approach. For extraction in methods, we will record study aim, design (RCT or observational study) and total study duration. For participants, we will extract the following data: total number of participants being randomised, age (mean or median), sex, ethnicity and comorbidities. Regarding intervention, we will record the name, dose, number of an intervention, method of administration (oral, intramuscular or intravenous injection) and cointerventions. Outcome measurements will be extracted as continuous or dichotomous. For continuous outcomes, we will record the name of an outcome, and mean and SD from the included trials (if the SD is not reported, we will calculate from 95% CI or SE); for dichotomous outcomes, we will record the numbers of events in the experimental group and the control group. Finally, the results of the included trials will be briefly summarised.

## Risk of bias assessment

Risk of bias of the included studies will be assessed using a tool developed by the Cochrane Collaboration. This tool includes six domains: random sequence generation, allocation concealment, blinding, incomplete outcome data, selective outcome reporting and other bias. The six domains will be separately evaluated and categorised as low, unclear or high risk of bias. Finally, overall quality of this systematic review will be summarised with GRADEpro (http://www.gradepro.org).

## Data analysis

A traditional meta-analysis will be performed to combine the effect size of each of the 6 SGLT2 inhibitors. Effect sizes of the 6 SGLT2 inhibitors will be compared separately in four situations: monotherapy, dual therapy, triple or quadruple therapy and in combination with insulin. Data of a SGLT2 inhibitor from different trials will only be combined if it is administered in the same dose. The effect sizes of the 6 SGLT2 inhibitors in continuous outcomes (the changes of HbA1c, body weight and blood pressure compared to baseline) will be calculated and synthesised by mean difference. If different units in a continuous outcome are used, we will use standardised mean difference (SMD) instead. The effect sizes of the SGLT2 inhibitors in dichotomous outcomes (the incidence of adverse events) will be calculated and combined with relative risk (RR). The 95% CIs of MD, SMD and RR will be calculated. If change-from-baseline values are not reported, we will calculate them from 95% CIs, SEs or individual variances. If these variables are not reported, we will calculate MD or SMD through the values reported at baseline and at the end of follow-up, assuming a correlation coefficient of 0.5 between the baseline and follow-up values. Before choosing fixed or random effect model to combine the effect size, we will run a heterogeneity test. The test will be performed by calculating I^2^ statistic, and an I^2^ >50% will be taken as important statistical heterogeneity.^36^ If an I^2^ >50% is found, we will perform a meta-regression to find potential confounders. A subgroup analysis will be subsequently performed according to the confounders (eg, if duration of diabetes is one of the confounders, we will recombine effect size of the SGLT2 inhibitors in patients with diabetes of short vs long duration). In subgroup analyses, if an I^2^ >50% is still found, we will not perform meta-analysis. Publication bias will be assessed with a funnel plot (to check if the plot is symmetrical) and the Egger's test (to check if there is a statistical significance). Furthermore, to reduce the impact of publication bias on the results, we will run a trim-and-fill analysis to find the best estimate for combining the effect size of each SGLT2 inhibitor.

After running the traditional meta-analysis, we will perform a NMA that compares all pairs of the SGLT2 inhibitors and their combinations (including monotherapy, dual therapy, triple therapy and quadruple therapy). The NMA will first be analysed through within-trial comparisons (direct comparisons) and then incorporated with indirect comparisons from two trials that use the same control. The NMA will be carried out under a frequentist framework developed with a graph-electrical network.^25^ This graph-electrical network accounts for correlated treatment effects in multiarm trials, which was not solved in a previous model within the frequentist framework.^24^ We will perform the NMA using the ‘netmeta’ package in R software (V.3.2.0, R Foundation for Statistical Computing, Vienna, Austria (http://www.r-project.org/)); the ‘netmeta’ package was developed according to graph-electrical network. After incorporating the direct and indirect comparisons in a network, we will use the ‘netrank’ function in the ‘netmeta’ package to generate a ranking of the SGLT2 inhibitors administered in different doses. We will report clinically relevant difference instead of statistical significance in all pairs of comparisons. Clinical superiority will be ascribed if any SGLT2 inhibitor improves HbA1c, body weight and blood pressure for at least 0.3%,^7^ 2.3 kg^37^ or 5 mm Hg,^38^ respectively, compared with the other SGLT2 inhibitors. The consistency of the network will be tested by a Cochrane's Q statistic for multivariate meta-analysis.^39^ This Q statistic can be decomposed in a sum of within-trial Q statistic and one between-trial Q statistic, which incorporate the design inconsistency.^40^ We will also use a net-heat plot to highlight inconsistency in the network. The net-heat plot is a matrix imaging that emphasises hot spots of inconsistency in the network and renders possible drivers. If performing quantitative synthesis is not possible, we will give a narrative review of the findings.

## Handling missing data

Scenarios of missing data are commonly encountered in data extraction. We will contact authors by email to ask for original data. If the original data are not available, we will try to compute them through other variables reported in the articles, for example, SD will be estimated from the 95% CI, p values or SEs.

## Sensitivity analysis

We will first exclude trials with high risk of bias to check if the results are consistent. Then we will exclude trials that are not published in peer-review journals to examine whether the data source influences the results. Second, we will exclude open-label trials and re-run the meta-analysis, since open-label trials are at high risk for performance bias. Third, we will report those trials sponsored by the manufacturers of the drugs, exclude them and run the meta-analysis again.

## Discussion

Several questions are answered in this protocol. First, why do we choose a network meta-analysis to study the relative effectiveness of the SGLT2 inhibitors? In traditional meta-analyses, only head-to-head comparisons can be combined, and indirect evidence will be ignored. However, whether one new drug is superior to another is interesting to clinicians as well as to patients. It is costly to perform a new trial to find the answer, so a network meta-analysis is beneficial in this situation for combining all the direct comparisons and simulating indirect comparisons without the need for new trials. Second, how can the reliability of the indirect comparisons be guaranteed? The indirect comparisons are simulated in such a way that treatments share the same control in two trials, so the coherence of the comparison loop is a major concern in performing NMA. We will use a generalised Cochrane's Q statistic to test the consistency of the network. If inconsistency exists, we will use a ‘leave-one-comparison-out’ approach to solve the problem. Third, why do we choose a frequentist method? Although most of the NMA analyses are performed using the Bayesian method, there is no consensus on which the best statistical solution is for running a NMA. We will choose the ‘netmeta’ function, a statistical method based on the frequentist framework, because it accounts for within-trial correlation by reweighting all comparisons in each multiarm trial, which has not been solved in other methods. Fourth, trials compared canagliflozin 100 mg with canagliflozin 300 mg, and empagliflozin 10 mg with empagliflozin 25 mg in patients who had not been on SGLT2 inhibitors before.^12^ ^18^ However, the larger doses should only be used in people who have tolerated the smaller doses but have not had an adequate effect on HbA1c, so people who do not respond adequately to the starting dose may be poor responders to SGLT2 inhibitors, and the effects of canagliflozin 300 and empagliflozin 25 may be less than those seen in the trials. To solve this problem, we will include trials of fixed dose combinations of an SGLT2 inhibitor with a DPP4 inhibitor (patients in these trials may also have been poor responders, leading to dual therapy use) or with other antihyperglycaemia medications. We will compare all monotherapy, dual therapy, triple or quadruple therapy in a network meta-analysis, through this method; we will thus observe which therapy achieves the best effect in poor responders—using larger doses or using combinations such as SGLT2 + DPP4.

In conclusion, we present a NMA protocol to assess the relative effectiveness and safety of SGLT2 inhibitors. The result of this NMA will be disseminated through a peer-review journal and conference abstracts.
